# Eating Alone is Differentially Associated with the Risk of Metabolic Syndrome in Korean Men and Women

**DOI:** 10.3390/ijerph15051020

**Published:** 2018-05-18

**Authors:** Chul-Kyoo Kim, Hyun-jin Kim, Hae-Kyung Chung, Dayeon Shin

**Affiliations:** 1 Department of Sociology, Korea University, Seoul 02841, Korea; ckkim@korea.ac.kr (C.-K.K.); combabe@naver.com (H.K.); 2 Department of Food and Nutrition, Hoseo University, Asan 31499, Korea; hkchung@hoseo.edu; 3 Department of Public Health, Food Studies and Nutrition, Syracuse University, Syracuse, NY 13244, USA

**Keywords:** eating alone, metabolic syndrome, obesity, Korea National Health and Nutrition Examination Survey (KNHANES)

## Abstract

Few studies have examined overall patterns of eating alone in relation to the risk of metabolic syndrome (MetS) in Korean populations. The present study aimed to examine the relationship between patterns of eating alone and the risk of MetS in Korean adults. Data from the Korea National Health and Nutrition Examination Survey (KNHANES) for 2013–2015 were used, with 8988 Korean adult participants, including 3624 men and 5364 women, aged 18 to 64 years. Patterns of eating alone were categorized into eight groups based on the total frequency of eating alone on a daily basis in the past one year: (1) three times for breakfast, lunch, and dinner; (2) twice for breakfast and dinner; (3) twice for lunch and dinner; (4) twice for breakfast and lunch; (5) once for breakfast only; (6) once for lunch only; (7) once for dinner only; and (8) never eating alone. The presence of MetS has been defined by the National Cholesterol Education Program Adult Treatment Panel III (NCEP ATP III) and International Diabetes Federation (IDF). Multivariable logistic regression analyses were performed to assess the association between patterns of eating alone versus the risk of MetS after controlling for age, income, occupation, number of family members, generation types, marital status, smoking status, and physical activity. The prevalence of MetS was the highest in men and women aged 40–64 who had breakfast, lunch, and dinner alone (50.1% and 36.8%, respectively). Men who had dinner alone or lunch and dinner alone compared with those who eat with others had a significantly higher risk of MetS, with adjusted odds ratios (AOR) of 1.51, and a 95% confidence interval (CI) of 1.06–2.16; and an AOR of 1.54, with a 95% CI of 1.05–2.25, respectively. Women who had breakfast alone compared with those who ate with others had a significantly lower risk of MetS (AOR 0.70, 95% CI 0.53–0.94). In conclusion, patterns of eating alone are differentially associated with the risk of MetS in a representative sample of Korean adults. Future studies are warranted to identify dietary patterns across the different eating alone patterns in relation to various health outcomes in Korean adult populations.

## 1. Introduction

Dietary behaviors have been rapidly changing in Korea, and the phenomenon of eating alone more often in Korean society has been receiving considerable attention [1]. Korean society has long been dominated by collectivism and community but has been experiencing the creation of an ‘alone generation’. ‘Alone generation’ or loner are terms that have been used since 2010 for Koreans who willingly do activities alone [2,3]. These individuals eat, drink, and travel alone. The increasing prevalence of eating alone can be partially explained by a demographic perspective of increased single-person households, which reached 27.9% in 2016 [4], as well as by a cultural perspective of the expansion of individualism [5,6,7]. Eating alone, however, is not simply a dietary pattern, but is an outcome reflecting the structural and cultural changes in Korean society, such as changes in social relationships, long working hours, and a deepening individualist culture.

In previous studies, Korean university students [8] and Japanese men [9] who eat alone compared to those who eat with others were more likely to display unhealthy eating behaviors such as high consumption of fast foods. Increased risks for depression or depressive symptoms were observed in Japanese [10,11] and Chinese older adults [12] who eat alone, and a lower quality of life as measured by the EuroQol five-dimension questionnaire (EQ-5D) was found in Korean adult men who eat alone compared to those who eat with a companion [13]. In a nationwide Thai Cohort Study, feeling unhappiness was significantly associated with frequent eating alone [14]. 

Metabolic syndrome (MetS) is the combination of the metabolic disturbances of obesity, insulin resistance, dyslipidemia, and hypertension [15]. An increasing prevalence of MetS has been found in Korean adults in recent years [16]. The factors associated with the increasing prevalence of MetS in Korean adults may be due to the high meat dietary patterns [17], sugar-sweetened beverage patterns [18], higher body mass index (BMI), and current smoking rates [19].

Korea is experiencing a dramatic increase in the number of single-person households [4]. Previous research focused on the impact of the total frequency of eating alone occasions on health outcomes, such as weight status [8,20], MetS [21], and depressive symptoms [12] or depression [10,22]. However, to the best of our knowledge, the impact of different combinations of eating alone patterns in terms of breakfast, lunch, or dinner on the risk of MetS in a representative sample of Korean adults has not yet been fully explored. The present study aimed to examine the relationship between patterns of eating alone and the risk of MetS in Korean adults. We hypothesized that the patterns of eating alone are differentially associated with the components of MetS in Korean adults.

## 2. Data and Methods

Data from the Korea National Health and Nutrition Examination Survey (KNHANES) for 2013–2015 were used for this study. KNHANES is an ongoing cross-sectional survey designed using complex, stratified, multistage, and probability cluster sampling to obtain nationally representative estimates. A total of 22,948 study participants completed the KNHANES 2013–2015 survey. Exclusion criteria for the study included those participants under 19 or 65 years or older (*n* = 9423), pregnant or lactating women (*n* = 237), those with daily energy intakes below 500 kcal and over 5000 kcal (*n* = 222), and those with missing responses on daily energy intake (*n* = 1724). We also excluded study participants with missing responses about socio-demographic characteristics (*n* = 1640), health-related behaviors such as smoking status and physical activity (*n* = 94), meal companionship (*n* = 45), components of MetS measurements (*n* = 568), and BMI (*n* = 7). The final analytic sample consists of 8988 participants (3624 men and 5364 women).

Determining a respondent’s meal companion during the last year was assessed using the question: “During the last year, did you usually eat breakfast with others?” If participants answered “no”, they were classified as “eating alone”. The “breakfast” meal occasion question was replaced with lunch or dinner. 

A total of eight categories were created based on the total frequency of eating alone on a daily basis in the past one year: (1) three times for breakfast, lunch, and dinner; (2) two times for breakfast and dinner; (3) two times for lunch and dinner; (4) two times for breakfast and lunch; (5) once for breakfast only; (6) once for lunch only; (7) once for dinner only; and (8) zero times eating alone.

The presence of MetS has been defined by the National Cholesterol Education Program Adult Treatment Panel III (NCEP ATP III) [23] and International Diabetes Federation (IDF) [24]. Individuals with three or more of the following risk factors were defined as having MetS: (1) abdominal obesity (waist circumference ≥ 90 cm for men and ≥ 80 cm for women; (2) elevated triglycerides (TG) (fasting TG ≥ 150 mg/dL), or individuals who had taken medications for dyslipidemia or had a diagnosis of dyslipidemia from a physician; (3) low high-density lipoprotein cholesterol (HDL-C) levels (fasting HDL-C < 40 mg/dL for men and < 50 mg/dL for women); (4) elevated fasting blood glucose (FBG) levels (FBG ≥ 100 mg/dL), or individuals who had taken medication for high blood glucose or had a diagnosis of diabetes from a physician; and (5) elevated blood pressure (BP) (systolic BP (SBP) ≥ 130 mmHg or diastolic BP (DBP) ≥ 85 mmHg), or individuals who had taken medication for high BP (HBP) or had a diagnosis of HBP from a physician. 

Sociodemographic characteristics were considered, such as age (in years, continuous), education (below or equivalent to elementary school graduates, middle school graduates, high school graduates or college graduates and above), marital status (married or unmarried), income (lower, lower middle, upper middle, or highest), number of family members in a household (1, 2, 3, 4, 5, or ≥ 6 members), and household type (single person, married couples without children, other (one generation), married couples with children, single parent with children, other (two generations), or more than three generations), along with health-related behaviors such as smoking status (nonsmoker, former smoker, current smoker) and physical activity (yes or no; yes to vigorous intensity activities for at least 75 min/week, or moderate intensity activities for at least 150 min/week, or an equivalent combination of moderate and vigorous intensity activity during a typical week).

Distribution of sociodemographic and lifestyle factors by sex were assessed by the frequency and weighted percentages. Distribution of sociodemographic and lifestyle characteristics based on the eight patterns of eating alone were computed and tested by Chi-square analyses. Mean and standard errors of anthropometric and biomarkers of MetS were calculated and then compared across the different patterns of eating alone by sex. Post-hoc Bonferroni tests were conducted to detect significant differences between each group. Adjusted odds ratios (AOR) and 95% confidence intervals (CI) were calculated to separately examine the association between the patterns of eating alone and the risk of MetS and its five components in men and women, after controlling for confounding variables. Lastly, the prevalence of MetS was calculated in the overall population stratified by different age groups (19–39 vs. 40–64 years) and sex (men vs. women). Sample weights, strata, and primary sampling units were applied to account for the complex survey design, survey non-response, and post-stratification design [25]. A two-tailed *p* value (< 0.05) was considered statistically significant. All statistical analyses were performed using SAS (version 9.4; SAS Institute, Inc., Cary, NC, USA).

## 3. Results

Distribution in age group, education, occupation, number of family members in a household, household generation type, marital status, physical activity, and smoking status significantly differed by sex (*p* value < 0.05) (Table 1). 

Patterns of eating alone significantly differed by age group, education, income status, occupation, number of family members, household generation type, and marital status in men (all *p* values < 0.05). In women, patterns of eating alone significantly differed by age group, education, income, occupation, number of family members, household generation type, marital status, and smoking status (all *p* values < 0.05) (Table 2). Men with more than a college education were more likely to eat with others (46.0%) or had only breakfast alone (50.1%). Married men had the highest proportion of zero times eating alone (74.0%). A higher proportion of adults with an elementary school degree who had breakfast, lunch, and dinner alone was found in both men and women (14.8% and 21.0%, respectively) compared with other patterns of eating alone. A higher proportion of employed men ate with a companion, whereas unemployed men were more likely to eat breakfast, lunch, and dinner alone (84.8% vs. 34.9%, respectively). Employed women had higher rates of eating breakfast and lunch alone (75.7%), and unemployed women had higher rates of eating breakfast and lunch alone (61.9%). More than 70% of women from two-generation households either never ate alone or only had breakfast alone. A higher number of married women had lunch alone (88.5%), breakfast and lunch alone (85.4%), and breakfast, lunch, and dinner alone (84.5%). Unmarried men had highest proportion of eating breakfast only and dinner only alone (32.3% and 32.2%, respectively).

Anthropometric measurements, components of MetS, and energy intake across the patterns of eating alone are presented in Table 3. In men, no significant difference was found except for energy intake. Men who had breakfast alone reported higher energy intakes compared to those who reported never eating alone, lunch alone, dinner alone, or lunch and dinner alone (all *p* values < 0.05). In women, mean and standard errors of BMI, waist circumference, SBP, DBP, total cholesterol, and triglycerides significantly differed with the patterns of eating alone (all *p* values < 0.05). BMI in women who reported eating breakfast, lunch, and dinner alone had significantly higher BMI compared to those who ate breakfast alone. Women who had breakfast, lunch, and dinner alone had a significantly higher waist circumference than those who ate all meals with others, breakfast alone, and breakfast and dinner alone, respectively. Women who had breakfast, lunch, and dinner alone had a significantly higher SBP compared with those who ate with others, ate breakfast alone, lunch alone, dinner alone, breakfast and lunch alone, and breakfast and dinner alone. Women who had breakfast, lunch, and dinner alone had a significantly higher DBP and total cholesterol level compared with those who ate with others and ate breakfast alone. Women who had breakfast alone had higher HDL-C compared with those who ate breakfast, lunch, and dinner alone. Women who ate breakfast, lunch, and dinner alone had a significantly higher level of TG compared with those who had breakfast and dinner alone, breakfast alone, and ate with others (Table 3).

Men who ate dinner alone or lunch and dinner alone compared with those who ate with others had a significantly higher risk of MetS (adjusted odds ratios (AOR) 1.51, 95% confidence intervals (CI) 1.06–2.16; AOR 1.54, 95% CI 1.05–2.25, respectively). Women who had breakfast alone compared with those who ate with others had a significantly lower risk of MetS (AOR 0.70, 95% CI 0.53–0.94). For abdominal obesity, men who had lunch and dinner alone, breakfast and dinner alone, or breakfast, lunch, and dinner alone all had a significantly higher risk of abdominal obesity (AOR 2.01, 95% CI 1.27–3.20; AOR 1.50, 95% CI 1.00–2.25; AOR 1.60, 95% CI 1.01–2.51, respectively) compared with those who ate with others. No significant association was found between patterns of eating alone and the risk of abdominal obesity in women. Women who had lunch and dinner alone compared with those who ate with others had a lower risk of low HDL-C (AOR 0.71, 95% CI 0.52–0.96). Women who had lunch alone had a higher risk of elevated TG (AOR 1.24, 95% CI 1.00–1.53) compared with those who ate with a companion. Women who had breakfast and dinner alone had a lower risk for elevated FBG compared with those who eat with others (AOR 0.59, 95% CI 0.38–0.92). In men, those who ate breakfast and dinner alone had higher risk of elevated BP compared with those who ate with others (AOR 1.54, 95% CI 1.03–0.30) (Table 4).

**Figure 1:**
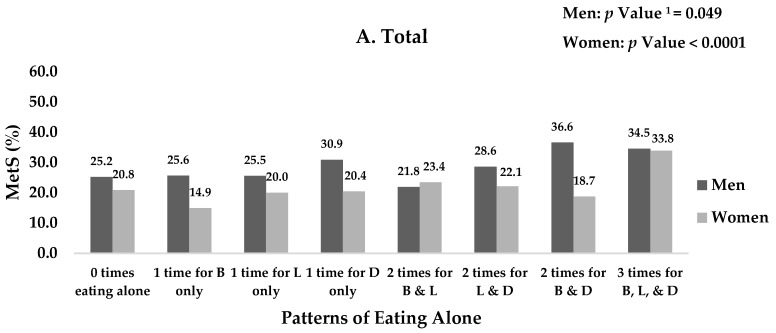

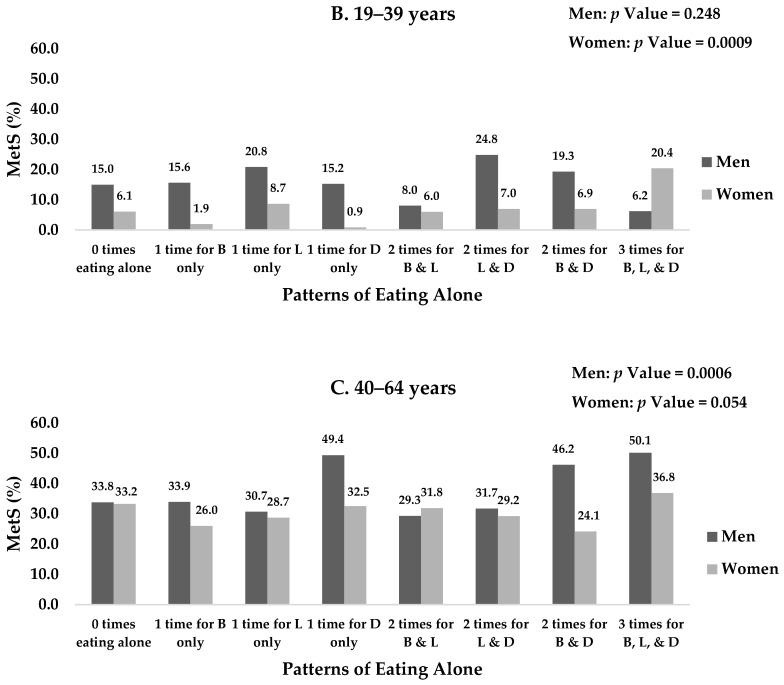
Prevalence of metabolic syndrome by patterns of eating alone by sex and age groups. ^1^ *p* Value is based on Chi-square test. MetS: Metabolic syndrome; B: Breakfast; L: Lunch; and D: Dinner.

A significant association was observed between the prevalence of MetS and the patterns of eating alone in men and women (all *p* values < 0.05). When stratified by age group (19–39 vs. 40–64 years), a significant relationship was found between the prevalence of MetS and the patterns of eating alone in women aged 19–39 years. In men aged 40–64 years, there was a significant association between the prevalence of MetS and the patterns of eating alone (*p* value = 0.0006). Notably, the prevalence of MetS was the highest (50.1%) in men aged 40–64 years who had breakfast, lunch, and dinner alone (Figure 1).

## 4. Discussion

The present study demonstrated that the patterns of eating alone are differentially associated with the risk of MetS in Korean men and women. We found that men who ate 1 time alone for dinner or two times alone for lunch and dinner in comparison with zero times of eating alone demonstrated a significantly higher odds of having MetS after controlling for numerous sociodemographic and lifestyle factors. In women, we found those who ate one time alone for breakfast had lower odds of developing MetS.

In the present study, age, education, income, occupation, number of family members in a household, household generation types, and marital status were significantly associated with patterns of eating alone in both men and women. In a study investigating various factors for eating alone for dinner among Korean adults [13], the authors found that age group, living arrangement, household income, having a spouse, and education level were all significantly associated with having a dinner companion. Specifically, the study found that men aged 50 years or older, with no spouse, smokers, and with a low self-care level had all significantly higher rates of eating alone for dinner. In the present study, similar finding was shown as men aged ≥ 50 years who live alone with lower income status were more likely eat two times alone for breakfast and dinner or eat three times alone for breakfast, lunch, and dinner.

Notably, men in one-person households were more likely eat lunch and dinner; breakfast and dinner; and breakfast, lunch, and dinner alone (23.0%, 20.8%, and 31.8%, respectively). Women in one-person households also had higher rates of eating breakfast, lunch, and dinner alone (22.7%). In Korea, single-person households have increased from 27.9% in 2016, being projected to reach 36.3% in 2045 [26]. Moreover, households headed by a single man or woman and single-person households were more likely to experience food insecurity in comparison with two-adult headed households in a Korean population [27]. The authors stated that the food insecurity status of households may be partially attributed to poorer economic status.

Unhealthy eating behaviors such as skipping meals and higher risk of obesity were found for older Japanese adults who ate alone in a single-person household [9]. The authors found that there are joint and combined effects of eating alone and living alone in terms of unhealthy dietary behaviors and unfavorable health outcomes. In the meantime, no significant association was found between Japanese older men who ate and lived alone and mortality risk in a fully-adjusted model that accounted for social adjustment [28]. Notably, men who ate alone yet lived with others had increased risk for mortality. This illustrates eating alone may be an independent risk factor for adverse health outcomes in men, regardless of living arrangements.

In the present study, men who had dinner alone and breakfast and dinner alone had an increased risk of MetS. Men who had dinner alone and breakfast and dinner alone were more likely to be in a single-person household (20.2% vs. 20.8%, respectively). Specifically, more than half the men who had dinner alone were unmarried (54.5%). This highlights that the high occurrence of eating alone may be an important risk factor for MetS in unmarried men as often women may promote the health of the men they live with through cooking, shopping, and menu planning. This parallels with previous findings that Korean adult men who ate alone without a spouse had a higher likelihood of having MetS in comparison to those men with a spouse [21]. In addition, married people demonstrate longer survival and lower mortality rates compared with those who have never married, or are separated, widowed, or divorced [29,30,31]. In another study investigating the association between marital status and vegetable intake among Koreans older than 30 years, married individuals consume more vegetables compared with other marital status groups [32], and vegetable intake was reported to be the lowest among those who had never married.

Men who had lunch and dinner alone, breakfast and dinner alone, and breakfast, lunch, and dinner alone had higher risks for abdominal obesity, but no significant association was found in women. When the total number of eating episodes was examined in relation to the risk of MetS in Korean adults [33], only men who eat less than or equal to twice per day had a significantly higher risk for abdominal obesity compared with those who eat three meals per day. Unhealthy meals including high fat intake and energy-dense food increased oxidative stress levels [34] of men who eat alone, which may explain the pathway to an increased risk of MetS in men [35]. Interestingly, in women, those who had breakfast alone had a decreased risk for MetS. We found that women who ate breakfast alone had a significantly lower BMI, waist circumference, SBP, DBP, TC, and TG levels compared with those who ate breakfast, lunch, and dinner alone. They are young age groups (19–29 years) with higher education levels (more than college graduate) and highest quartile income level, and mostly living in a two-generation household. In the meantime, women who had all meals alone had the highest BMI, waist circumference, SBP, DBP, total cholesterol, and TG. This indicates the highest metabolic risks for those women who ate alone three times each day. 

Sobal and Nelson [6] suggested that structural individualism is related to social isolation in the postindustrial era and that it has acted as a barrier to eating with others. Eating alone does not simply reflect dietary behavior; it can also be viewed as an indication of social and cultural changes, such as rising levels of individualism, more social isolation, and increasing loneliness. In the past in Korea, food was a medium for sharing affection among people [36]. Food can satisfy hunger, but it also has critical values, such as building social ties and connections among people [6,37].

The strengths of this study include using a nation-wide, representative sample of the Korean population and its generalizability to Korean adult population. To the best of our knowledge, this is the first study to identify different patterns of eating alone in Korean adults. Previous studies did not account for different eating alone patterns based on meal occasions rather than the total number of meals in relation to socioeconomic and lifestyle factors and MetS. Furthermore, the present study controlled for a number of confounders including socioeconomic status, household composition, generation types, and physical activity. Despite the strengths of this study, the study has the following limitations. Using self-reported questionnaires, recall bias may have occurred. Secondly, the KNHANES is a cross-sectional study [25], so the cause-effect relationship cannot be established, although the association or relationship between patterns of eating alone and the risk of MetS was observed. Furthermore, eating alone for each meal occasion was obtained from a simplified question that can be only answered with two responses, so in-depth information such as the reasons for eating alone was not available in this study.

## 5. Conclusions 

Our study findings highlight that the patterns of eating alone are differentially associated with sociodemographic and lifestyle factors, and the risk of MetS with sex differences in Korean men and women. Men who had dinner alone or breakfast and dinner alone had an increased risk of MetS after adjusting for age, income, occupation, number of family members in a household, generation type, marital status, smoking status, and physical activity. Women who had breakfast alone had a decreased risk of MetS after controlling for covariates. Future studies are warranted to identify distinct dietary patterns across the different types of eating alone in relation to various health outcomes in the Korean adult population.

## Figures and Tables

**Table 1:** Sociodemographic and lifestyle characteristics of study participants.

Sociodemographic and Lifestyle Characteristics	Men		Women		Total	*p* Value ^1^
	*n*	(%)	*n*	(%)	*n*	
Age (years)						
19–29	607	(23.0)	755	(20.3)	1362	<0.0001
30–39	740	(22.5)	1122	(21.1)	1862	
40–49	859	(25.2)	1366	(25.8)	2225	
50–59	950	(23.4)	1481	(24.6)	2431	
60–64	468	(5.9)	640	(8.3)	1108	
Education						
≤Elementary School	299	(5.8)	692	(10.2)	991	<0.0001
Middle School Graduate	319	(7.1)	575	(9.7)	894	
High School Graduate	1444	(42.7)	2110	(40.8)	3554	
≥College Graduate	1562	(44.4)	1987	(39.3)	3549	
Income						
Highest	927	(25.4)	1385	(26.1)	2312	0.3706
Upper Middle	902	(24.6)	1384	(25.5)	2286	
Lower Middle	945	(26.2)	1332	(24.7)	2277	
Lowest	850	(23.9)	1263	(23.7)	2113	
Occupation						
Employed	2990	(81.4)	3062	(57.3)	6052	<0.0001
Unemployed	634	(18.6)	2302	(42.7)	2936	
No. of Family Members in a Household						
1	256	(7.1)	277	(4.5)	533	<0.0001
2	783	(18.0)	1253	(20.5)	2036	
3	1031	(29.3)	1536	(30.2)	2567	
4	1141	(33.8)	1626	(31.7)	2767	
5	315	(9.1)	498	(9.9)	813	
≥6	98	(2.7)	174	(3.2)	272	
Household Generation Types						
Single-Person Household	256	(7.1)	277	(4.5)	533	<0.0001
1 Generation Households	641	(14.1)	954	(15.2)	1595	
2 Generations Households	2442	(71.1)	3636	(71.2)	6078	
≥3 Generations Households	285	(7.7)	497	(9.1)	782	
Marital Status						
Married	2759	(69.3)	4501	(78.5)	7260	<0.0001
Unmarried	865	(30.7)	863	(21.5)	1728	
Physical Activity						
Yes	1596	(47.2)	2095	(41.5)	3691	<0.0001
No	2028	(52.8)	3269	(58.5)	5297	
Smoking Status						
Nonsmoker	989	(29.8)	4894	(90.7)	5883	<0.0001
Former Smoker	1142	(28.1)	214	(4.3)	1356	
Current Smoker	1493	(42.1)	256	(5.0)	1749	

^1^*p* Value is based on Chi-square test.

**Table 2:** Sociodemographic and lifestyle characteristics across the patterns of eating alone in men and women.

**Men**	**0 Times** **Eating Alone** **(*n* = 1975)**		**1 Time for** **B Only** **(*n* = 572)**		**1 Time for** **L Only** **(*n* = 312)**		**1 Time for** **D Only** **(*n* = 192)**		**2 Times for** **B & L** **(*n* = 127)**		**2 Times for** **L & D** **(*n* = 133)**		**2 Times for** **B & D** **(*n* = 163)**		**3 Times for** **B, L, & D** **(*n* = 150)**		***p* Value ^1^**
	***n***	**(%)**	***n***	**(%)**	***n***	**(%)**	***n***	**(%)**	***n***	**(%)**	***n***	**(%)**	***n***	**(%)**	***n***	**(%)**	
Age (years)																	
19–29	303	(20.6)	87	(20.8)	71	(32.7)	45	(32.0)	22	(24.5)	34	(34.9)	22	(18.3)	23	(23.0)	<0.0001
30–39	457	(25.2)	125	(24.4)	55	(19.7)	40	(22.2)	13	(10.4)	13	(10.7)	24	(17.4)	13	(12.5)	
40–49	505	(27.4)	154	(26.7)	55	(18.2)	44	(23.3)	30	(29.4)	24	(20.5)	30	(19.7)	17	(14.1)	
50–59	481	(21.7)	146	(23.8)	77	(21.2)	47	(19.0)	34	(25.5)	47	(28.6)	64	(37.8)	54	(33.5)	
60–64	229	(5.2)	60	(4.4)	54	(8.3)	16	(3.4)	28	(10.2)	15	(5.2)	23	(6.8)	43	(16.9)	
Education																	
≤Elementary School	137	(4.6)	31	(4.0)	32	(7.6)	19	(6.4)	13	(7.9)	17	(9.0)	18	(9.5)	32	(14.8)	<0.0001
Middle School Graduate	158	(6.5)	45	(6.5)	25	(5.3)	13	(5.7)	18	(12.3)	27	(15.8)	10	(5.9)	23	(12.6)	
High School Graduate	793	(43.0)	211	(39.3)	130	(45.8)	81	(44.6)	46	(37.0)	51	(44.1)	79	(49.7)	53	(38.2)	
≥College Graduate	887	(46.0)	285	(50.1)	125	(41.3)	79	(43.3)	50	(42.8)	38	(31.1)	56	(35.0)	42	(34.4)	
Income																	
Highest	525	(26.9)	170	(28.7)	66	(19.5)	48	(25.7)	25	(20.4)	21	(16.8)	48	(24.8)	24	(14.7)	<0.0001
Upper Middle	512	(25.6)	156	(26.4)	73	(23.0)	39	(16.6)	29	(24.1)	28	(21.1)	41	(27.0)	24	(19.0)	
Lower Middle	496	(24.5)	164	(30.4)	83	(25.8)	52	(29.7)	34	(25.2)	37	(27.5)	38	(24.3)	41	(30.2)	
Lowest	442	(23.0)	82	(14.5)	90	(31.7)	53	(27.9)	39	(30.3)	47	(34.6)	36	(23.9)	61	(36.1)	
Occupation																	
Employed	1691	(84.8)	513	(88.9)	213	(66.7)	162	(81.5)	89	(66.3)	90	(63.7)	135	(81.6)	97	(65.1)	<0.0001
Unemployed	284	(15.2)	59	(11.1)	99	(33.3)	30	(18.5)	38	(33.7)	43	(36.3)	28	(18.4)	53	(34.9)	
No. of Family Members in a Household																	
1	62	(3.5)	20	(3.3)	12	(4.3)	37	(20.2)	4	(2.6)	30	(23.0)	34	(20.8)	57	(31.8)	<0.0001
2	444	(18.0)	103	(15.4)	77	(20.6)	51	(26.3)	30	(20.8)	24	(14.4)	30	(15.7)	24	(14.3)	
3	585	(30.5)	177	(32.9)	92	(30.8)	33	(17.4)	38	(31.0)	29	(19.2)	44	(27.0)	33	(24.7)	
4	647	(35.2)	189	(33.9)	101	(35.3)	59	(30.5)	39	(32.9)	43	(36.6)	38	(26.2)	25	(20.7)	
5	181	(9.8)	65	(11.6)	22	(6.6)	9	(4.0)	12	(10.2)	6	(6.1)	12	(7.9)	8	(5.8)	
≥6	56	(2.9)	18	(2.9)	8	(2.4)	3	(1.7)	4	(2.5)	1	(0.7)	5	(2.5)	3	(2.8)	
Household Generation Types																	
Single-Person Household	62	(3.5)	20	(3.3)	12	(4.3)	37	(20.2)	4	(2.6)	30	(23.0)	34	(20.8)	57	(31.8)	<0.0001
1 Generation Households	382	(15.3)	89	(12.9)	60	(14.6)	29	(12.8)	22	(15.8)	19	(11.1)	21	(9.2)	19	(10.8)	
2 Generations Households	1364	(72.6)	413	(75.4)	224	(77.1)	112	(60.9)	90	(75.3)	75	(57.5)	101	(65.3)	63	(50.0)	
≥3 Generations Households	167	(8.5)	50	(8.4)	16	(3.9)	14	(6.1)	11	(6.4)	9	(8.4)	7	(4.7)	11	(7.5)	
Marital Status																	
Married	1568	(74.0)	461	(74.5)	219	(60.6)	108	(45.5)	98	(70.1)	83	(52.4)	118	(64.1)	104	(57.7)	<0.0001
Unmarried	407	(26.0)	111	(25.5)	93	(39.4)	84	(54.5)	29	(29.9)	50	(47.6)	45	(35.9)	46	(42.3)	
Smoking																	
Nonsmoker	530	(29.0)	176	(33.6)	73	(26.0)	51	(28.1)	39	(33.1)	35	(28.2)	51	(35.9)	34	(26.9)	0.0715
Former Smoker	613	(28.3)	188	(30.4)	108	(29.3)	47	(19.3)	40	(27.1)	42	(27.9)	52	(24.0)	52	(32.2)	
Current Smoker	832	(42.7)	208	(36.0)	131	(44.7)	94	(52.6)	48	(39.8)	56	(43.9)	60	(40.0)	64	(40.8)	
Physical Activity																	
Yes	847	(45.9)	276	(51.1)	131	(45.9)	91	(49.5)	58	(49.3)	60	(48.5)	73	(48.2)	60	(43.5)	0.6344
No	1128	(54.1)	296	(48.9)	181	(54.1)	101	(50.5)	69	(50.7)	73	(51.5)	90	(51.8)	90	(56.5)	
Total	1975	(100.0)	572	(100.0)	312	(100.0)	192	(100.0)	127	(100.0)	133	(100.0)	163	(100.0)	150	(100.0)	
**Women**	**0 Times** **Eating Alone** **(*n* = 2360)**		**1 Time for** **B Only** **(*n* = 584)**		**1 Time for** **L Only** **(*n* = 1004)**		**1 Time for** **D Only** **(*n* = 251)**		**2 Times for** **B & L** **(*n* = 406)**		**2 Times for** **L & D** **(*n* = 255)**		**2 Times for** **B & D** **(*n* = 234)**		**3 Times for B, L, & D** **(*n* = 270)**		***p* Value ^1^**
	***n***	**(%)**	***n***	**(%)**	***n***	**(%)**	***n***	**(%)**	***n***	**(%)**	***n***	**(%)**	***n***	**(%)**	***n***	**(%)**	
Age (years)																	
19–29	371	(23.0)	119	(27.5)	88	(13.6)	46	(23.9)	42	(13.9)	36	(19.2)	36	(22.5)	17	(10.0)	<0.0001
30–39	537	(22.8)	118	(18.7)	292	(29.7)	30	(14.5)	81	(18.7)	26	(12.6)	21	(9.1)	17	(7.9)	
40–49	601	(25.3)	145	(24.8)	283	(28.2)	56	(21.1)	110	(30.4)	51	(19.1)	71	(31.2)	49	(21.3)	
50–59	573	(20.9)	155	(23.9)	249	(21.7)	94	(33.4)	119	(27.5)	95	(34.7)	83	(31.7)	113	(40.7)	
60–64	278	(8.0)	47	(5.2)	92	(6.8)	25	(7.1)	54	(9.5)	47	(14.3)	23	(5.4)	74	(20.0)	
Education																	
≤Elementary School	305	(10.1)	47	(7.0)	93	(6.9)	36	(12.3)	55	(11.9)	48	(15.3)	35	(10.1)	73	(21.0)	<0.0001
Middle School Graduate	241	(9.0)	55	(7.8)	90	(8.6)	28	(11.8)	43	(9.6)	36	(12.8)	35	(12.0)	47	(18.5)	
High School Graduate	926	(40.8)	247	(42.8)	409	(42.3)	100	(38.1)	158	(41.1)	106	(41.4)	83	(38.5)	81	(32.6)	
≥College Graduate	888	(40.1)	235	(42.4)	412	(42.2)	87	(37.7)	150	(37.4)	65	(30.5)	81	(39.3)	69	(28.0)	
Income																	
Highest	597	(25.7)	185	(32.6)	241	(22.9)	64	(23.2)	101	(24.1)	68	(28.8)	64	(28.8)	65	(28.7)	<0.0001
Upper Middle	611	(25.5)	153	(25.7)	276	(26.6)	65	(27.6)	119	(28.8)	63	(22.6)	51	(21.9)	46	(17.5)	
Lower Middle	569	(24.1)	143	(23.1)	254	(25.8)	84	(34.2)	105	(26.5)	52	(19.5)	61	(25.5)	64	(21.0)	
Lowest	583	(24.7)	103	(18.7)	233	(24.6)	38	(15.1)	81	(20.6)	72	(29.1)	58	(23.8)	95	(32.8)	
Occupation																	
Employed	1530	(64.9)	392	(67.0)	383	(39.2)	181	(72.1)	153	(38.1)	115	(43.0)	174	(75.7)	134	(49.0)	<0.0001
Unemployed	830	(35.1)	192	(33.0)	621	(60.8)	70	(27.9)	253	(61.9)	140	(57.0)	60	(24.3)	136	(51.0)	
No. of Family Members in a Household																	
1	38	(1.8)	23	(3.6)	14	(1.6)	30	(11.2)	13	(2.4)	28	(8.6)	52	(17.7)	79	(22.7)	<0.0001
2	541	(19.4)	107	(16.8)	197	(17.0)	80	(28.7)	90	(20.4)	86	(30.3)	70	(27.8)	82	(28.5)	
3	676	(30.1)	164	(29.6)	314	(32.2)	63	(28.0)	118	(29.7)	81	(36.2)	57	(25.2)	63	(26.4)	
4	742	(32.7)	201	(34.4)	374	(38.8)	56	(24.1)	134	(33.8)	45	(18.9)	42	(20.7)	32	(16.6)	
5	256	(11.5)	65	(11.7)	86	(8.8)	16	(5.9)	42	(11.3)	14	(5.5)	10	(7.0)	9	(4.6)	
≥6	107	(4.6)	24	(3.7)	19	(1.7)	6	(2.2)	9	(2.5)	1	(0.6)	3	(1.6)	5	(1.3)	
Household Generation Types																	
Single-Person Household	38	(1.8)	23	(3.6)	14	(1.6)	30	(11.2)	13	(2.4)	28	(8.6)	52	(17.7)	79	(22.7)	<0.0001
1 Generation Households	464	(16.2)	71	(11.5)	153	(12.6)	54	(20.5)	68	(15.1)	62	(21.0)	37	(13.7)	45	(15.0)	
2 Generations Households	1584	(70.4)	434	(76.3)	759	(78.2)	148	(60.8)	292	(74.4)	151	(64.6)	136	(64.6)	132	(56.4)	
≥3 Generations Households	274	(11.5)	56	(8.6)	78	(7.6)	19	(7.6)	33	(8.1)	14	(5.8)	9	(4.0)	14	(5.9)	
Marital Status																	
Married	1962	(76.9)	437	(67.7)	922	(88.5)	187	(67.8)	359	(85.4)	219	(81.6)	178	(70.4)	237	(84.5)	<0.0001
Unmarried	398	(23.1)	147	(32.3)	82	(11.5)	64	(32.2)	47	(14.6)	36	(18.4)	56	(29.6)	33	(15.5)	
Smoking																	
Nonsmoker	2148	(90.0)	539	(92.1)	905	(89.0)	228	(89.8)	387	(95.7)	235	(91.3)	210	(91.1)	242	(91.2)	0.0269
Former Smoker	94	(4.3)	18	(3.1)	50	(5.7)	7	(3.8)	11	(3.0)	10	(4.2)	15	(6.4)	9	(2.6)	
Current Smoker	118	(5.7)	27	(4.8)	49	(5.3)	16	(6.4)	8	(1.3)	10	(4.5)	9	(2.5)	19	(6.2)	
Physical Activity																	
Yes	885	(39.9)	233	(43.2)	407	(42.1)	92	(39.9)	164	(42.3)	102	(41.3)	106	(48.3)	106	(44.2)	0.4743
No	1475	(60.1)	351	(56.8)	597	(57.9)	159	(60.1)	242	(57.7)	153	(58.7)	128	(51.7)	164	(55.8)	
Total	2360	(100.0)	584	(100.0)	1004	(100.0)	251	(100.0)	406	(100.0)	255	(100.0)	234	(100.0)	270	(100.0)	

B: Breakfast; L: Lunch; and D: Dinner. ^1^ *p* Value is based on Chi-square test.

**Table 3:** BMI, components of metabolic syndrome, and energy intake by the patterns of eating alone in men and women.

**Men**	**0 Times Eating Alone** **(*n* = 1975)**		**1 Time for B Only** **(*n* = 572)**		**1 Time for L Only** **(*n* = 312)**		**1 Time for D Only** **(*n* = 192)**		**2 Times for B & L** **(*n* = 127)**		**2 Times for L & D** **(*n* = 133)**		**2 Times for B & D** **(*n* = 163)**		**3 Times for B, L, & D** **(*n* = 150)**	
	**Mean**	**(SE)**	**Mean**	**(SE)**	**Mean**	**(SE)**	**Mean**	**(SE)**	**Mean**	**(SE)**	**Mean**	**(SE)**	**Mean**	**(SE)**	**Mean**	**(SE)**
BMI (kg/m^2^)	24.4	(0.1)	24.6	(0.2)	24.6	(0.2)	24.5	(0.3)	24.2	(0.4)	25.4	(0.5)	24.8	(0.3)	24.4	(0.3)
WC (cm)	84.5	(0.2)	85.0	(0.4)	85.0	(0.6)	84.4	(0.8)	83.6	(1.0)	87.7	(1.1)	86.3	(0.8)	85.4	(0.9)
SBP (mmHg)	117.4	(0.3)	117.4	(0.6)	117.3	(0.9)	117.7	(0.8)	119.4	(1.6)	118.8	(1.2)	120.3	(1.5)	120.0	(1.3)
DBP (mmHg)	78.5	(0.3)	78.5	(0.4)	78.3	(0.6)	78.6	(0.7)	78.5	(1.1)	79.8	(1.1)	79.6	(1.0)	78.5	(1.1)
FBG (mg/dL)	99.5	(0.5)	99.6	(0.9)	97.7	(1.1)	101.3	(2.3)	97.2	(1.7)	101.0	(2.2)	103.5	(2.3)	103.7	(2.5)
Total Cholesterol (mg/dL)	190.2	(0.9)	190.7	(1.7)	188.5	(2.2)	185.1	(2.6)	186.7	(4.0)	190.8	(3.3)	186.5	(3.0)	182.0	(3.3)
HDL-C (mg/dL)	47.4	(0.3)	48.3	(0.5)	47.7	(0.7)	46.9	(0.8)	48.6	(1.0)	46.0	(1.0)	46.6	(0.9)	47.3	(1.0)
TG (mg/dL)	168.8	(3.7)	163.9	(5.6)	152.1	(8.8)	166.7	(13.1)	149.1	(10.2)	188.3	(15.8)	175.1	(13.0)	154.0	(8.7)
Energy Intake (kcal/d)	2467 ^b^	(21)	2663 ^a^	(43)	2366 ^b^	(57)	2262 ^b^	(64)	2420 ^a,b^	(92)	2308 ^b,c^	(92)	2451 ^a,b^	(75)	2422 ^a,b^	(103)
**Women**	**0 Times Eating Alone** **(*n* = 2360)**		**1 Time for B Only** **(*n* = 584)**		**1 Time for L Only** **(*n* = 1004)**		**1 Time for D Only** **(*n* = 251)**		**2 Times for B & L** **(*n* = 406)**		**2 Times for L & D** **(*n* = 255)**		**2 Times for B & D** **(*n* = 234)**		**3 Times for B, L, & D** **(*n* = 270)**	
	**Mean**	**(SE)**	**Mean**	**(SE)**	**Mean**	**(SE)**	**Mean**	**(SE)**	**Mean**	**(SE)**	**Mean**	**(SE)**	**Mean**	**(SE)**	**Mean**	**(SE)**
BMI (kg/m^2^)	23.0 ^a,b^	(0.1)	22.6 ^b^	(0.2)	23.0 ^a,b^	(0.1)	23.3 ^a,b^	(0.3)	22.8 ^a,b^	(0.2)	23.3 ^a,b^	(0.3)	22.6 ^a,b^	(0.2)	23.6 ^a^	(0.3)
WC (cm)	76.5 ^b^	(0.2)	74.9 ^c^	(0.4)	76.8 ^a,b^	(0.4)	76.9 ^a,b^	(0.7)	76.8 ^a,b^	(0.5)	77.0 ^a,b^	(0.8)	75.5 ^b,c^	(0.6)	78.8 ^a^	(0.7)
SBP (mmHg)	111.1 ^b^	(0.4)	109.5 ^b^	(0.7)	110.7 ^b^	(0.5)	110.7 ^b^	(1.0)	111.1 ^b^	(0.8)	112.6 ^a,b^	(1.1)	111.1 ^b,c^	(1.0)	116.5 ^a^	(1.2)
DBP (mmHg)	72.5 ^b^	(0.3)	72.2 ^b^	(0.5)	72.9 ^a,b^	(0.3)	72.3 ^a,b^	(0.7)	73.0 ^a,b^	(0.5)	73.1 ^a,b^	(0.7)	73.5 ^a,b^	(0.7)	75.1 ^a^	(0.7)
FBG (mg/dL)	95.2	(0.4)	94.1	(0.7)	95.3	(0.9)	94.8	(1.1)	95.7	(1.0)	95.1	(1.6)	92.9	(1.0)	98.4	(1.5)
Total Cholesterol (mg/dL)	185.8 ^b,c^	(0.8)	183.0 ^c^	(1.4)	189.0 ^a,b^	(1.2)	191.6 ^a,b^	(2.5)	189.8 ^a,b^	(1.9)	191.6 ^a,b^	(2.3)	188.7 ^a,b^	(2.6)	194.9 ^a^	(2.5)
HDL-C (mg/dL)	55.3 ^a,b^	(0.3)	56.6 ^a^	(0.6)	55.2 ^a,b^	(0.4)	55.2 ^a,b^	(0.8)	55.5 ^a,b^	(0.7)	55.4 ^a,b^	(0.8)	55.2 ^a,b^	(0.8)	53.3 ^b,c^	(0.8)
TG (mg/dL)	101.9 ^b,c^	(1.6)	94.0 ^c^	(2.8)	109.9 ^a,b^	(2.7)	105.9 ^a,c^	(5.3)	110.2 ^a,c^	(4.7)	110.1 ^a,b^	(5.1)	102.3 ^b,c^	(4.3)	129.8 ^a^	(6.5)
Energy Intake (kcal/d)	1793	(15)	1853	(33)	1781	(22)	1800	(44)	1821	(43)	1774	(47)	1859	(59)	1798	(49)

Bonferroni post-hoc test was conducted to test the differences between groups in patterns of eating alone. ^a,b,c^ Different letters, within a row, denote the significant differences between each group (*p* value < 0.05). SE: standard error; BMI: body mass index; WC: waist circumference; SBP: systolic blood pressure; DBP: diastolic blood pressure; FBG: fasting blood glucose; HDL-C: HDL-cholesterol; TG: triglycerides; B: Breakfast; L: Lunch; and D: Dinner.

**Table 4:** Adjusted odds ratios for the risk of metabolic syndrome and its components by the patterns of eating alone in men and women.

Patterns of Eating Alone	Men		Women	
	AOR ^1^	(95% CI)	AOR ^1^	(95% CI)
**Metabolic Syndrome**				
0 times eating alone	1.00	(Reference)	1.00	(Reference)
1 time for B only	1.04	(0.82–1.33)	0.70	(0.53–0.94)
1 time for L only	1.06	(0.75–1.49)	0.93	(0.75–1.14)
1 time for D only	1.51	(1.06–2.16)	0.84	(0.57–1.24)
2 times for B & L	0.75	(0.46–1.20)	0.94	(0.69–1.27)
2 times for L & D	1.26	(0.68–2.33)	0.70	(0.47–1.06)
2 times for B & D	1.54	(1.05–2.25)	0.77	(0.49–1.23)
3 times for B, L, & D	1.30	(0.83–2.03)	1.02	(0.70–1.47)
**Abdominal Obesity**				
0 times eating alone	1.00	(Reference)	1.00	(Reference)
1 time for B only	1.13	(0.90–1.43)	0.85	(0.67–1.07)
1 time for L only	1.19	(0.88–1.59)	0.97	(0.80–1.17)
1 time for D only	1.16	(0.81–1.66)	1.20	(0.88–1.65)
2 times for B & L	0.78	(0.48–1.26)	0.98	(0.74–1.30)
2 times for L & D	2.01	(1.27–3.20)	0.74	(0.52–1.03)
2 times for B & D	1.50	(1.00–2.25)	0.79	(0.54–1.15)
3 times for B, L, & D	1.60	(1.01–2.51)	1.12	(0.81–1.55)
**Low HDL-C**				
0 times eating alone	1.00	(Reference)	1.00	(Reference)
1 time for B only	0.98	(0.76–1.27)	0.86	(0.69–1.06)
1 time for L only	0.78	(0.57–1.08)	0.98	(0.81–1.18)
1 time for D only	1.23	(0.86–1.75)	1.07	(0.77–1.48)
2 times for B & L	0.66	(0.40–1.08)	0.87	(0.67–1.12)
2 times for L & D	1.35	(0.84–2.16)	0.71	(0.52–0.96)
2 times for B & D	0.86	(0.57–1.30)	1.07	(0.75–1.53)
3 times for B, L, & D	0.73	(0.45–1.18)	0.95	(0.68–1.31)
**Elevated TG**				
0 times eating alone	1.00	(Reference)	1.00	(Reference)
1 time for B only	1.09	(0.88–1.34)	0.85	(0.66–1.10)
1 time for L only	0.86	(0.64–1.16)	1.24	(1.00–1.53)
1 time for D only	1.19	(0.84–1.70)	0.88	(0.62–1.25)
2 times for B & L	0.90	(0.59–1.35)	1.05	(0.78–1.42)
2 times for L & D	1.17	(0.78–1.77)	1.02	(0.71–1.46)
2 times for B & D	1.27	(0.87–1.86)	1.03	(0.69–1.54)
3 times for B, L, & D	1.27	(0.81–1.98)	1.25	(0.89–1.77)
**Elevated FBG**				
0 times eating alone	1.00	(Reference)	1.00	(Reference)
1 time for B only	1.08	(0.86–1.34)	0.85	(0.65–1.11)
1 time for L only	0.95	(0.68–1.32)	0.85	(0.69–1.05)
1 time for D only	1.19	(0.83–1.71)	1.01	(0.70–1.47)
2 times for B & L	0.78	(0.49–1.24)	0.93	(0.70–1.24)
2 times for L & D	1.00	(0.63–1.56)	0.79	(0.54–1.15)
2 times for B & D	1.24	(0.85–1.81)	0.59	(0.38–0.92)
3 times for B, L, & D	1.19	(0.79–1.78)	0.73	(0.51–1.05)
**Elevated BP**				
0 times eating alone	1.00	(Reference)	1.00	(Reference)
1 time for B only	1.01	(0.80–1.28)	0.84	(0.64–1.09)
1 time for L only	1.22	(0.90–1.64)	0.82	(0.65–1.05)
1 time for D only	1.30	(0.92–1.83)	0.97	(0.68–1.40)
2 times for B & L	1.08	(0.73–1.61)	0.91	(0.65–1.26)
2 times for L & D	1.46	(0.90–2.36)	0.96	(0.65–1.41)
2 times for B & D	1.54	(1.03–2.30)	1.11	(0.72–1.70)
3 times for B, L, & D	1.13	(0.74–1.71)	0.84	(0.59–1.20)

^1^ Adjusted for age, income, occupation, number of family, generation types, marital status, smoking status, and physical activity. AOR: adjusted odds ratios; B: Breakfast; L: Lunch; D: Dinner; HDL-C: HDL-cholesterol; TG: triglycerides; FBG: fasting blood glucose; and BP: blood pressure.
